# Fractal Analysis of a Non-Newtonian Fluid Flow in a Rough-Walled Pipe

**DOI:** 10.3390/ma15103700

**Published:** 2022-05-22

**Authors:** Abdellah Bouchendouka, Zine El Abiddine Fellah, Zakaria Larbi, Zineeddine Louna, Erick Ogam, Mohamed Fellah, Claude Depollier

**Affiliations:** 1 Laboratory of Theoretical and Applied Fluid Mechanics LMFTA, Faculty of Physics, University of Sciences and Technology Houari Boumediene USTHB, BP 32 El Alia, Bab Ezzouar 16111, Algeria; abdourami27@yahoo.fr (A.B.); larbzak@gmail.com (Z.L.); lounazineeddine@gmail.com (Z.L.); 2 Laboratory of Mechanics and Acoustics, French National Center for Scientific Research CNRS LMA, UMR 7031, Centrale Marseille, Aix-Marseille University, CEDEX 20, F-13402 Marseille, France; ogam@lma.cnrs-mrs.fr; 3 Laboratory of Theoretical Physics, Faculty of Physics, University of Sciences and Technology Houari Boumediene USTHB, BP 32 El Alia, Bab Ezzouar 16111, Algeria; fellahm1@yahoo.fr; 4 Laboratory of Acoustics (LAUM), UMR 6613 CNRS, Acoustics Institute, Graduate School (IA-GS), Le Mans University, Avenue Olivier Messiaen, CEDEX 09, F-72085 Le Mans, France; claude.depollier@univ-lemans.fr

**Keywords:** fractal surface, surface fractal dimensions, non-Newtonian fluids, rough surface

## Abstract

The fully developed laminar flow of a viscous non-Newtonian fluid in a rough-walled pipe is considered. The fluid rheology is described by the power–law model (covering shear thinning, Newtonian, and shear thickening fluids). The rough surface of the pipe is considered to be fractal, and the surface roughness is measured using surface fractal dimensions. The main focus of this study lies in the theoretical investigation of the influence of the pipe surface roughness on the velocity profile and the Darcy friction factor of an incompressible non-Newtonian fluid. The plotted results demonstrate that shear thinning fluids are the most sensitive to the surface roughness compared with Newtonian and shear thickening fluids. For a particular value of the surface fractal dimension, there exists an intersection point where shear thinning, Newtonian, and shear thickening fluids behave the same way regarding the amplitude of the velocity profile and the friction factor. This approach has a variety of potential applications, for instance fluid dynamics in hydrology, blood flow in the cardiovascular system, and many industrial applications.

## 1. Introduction

**Figure 1:**
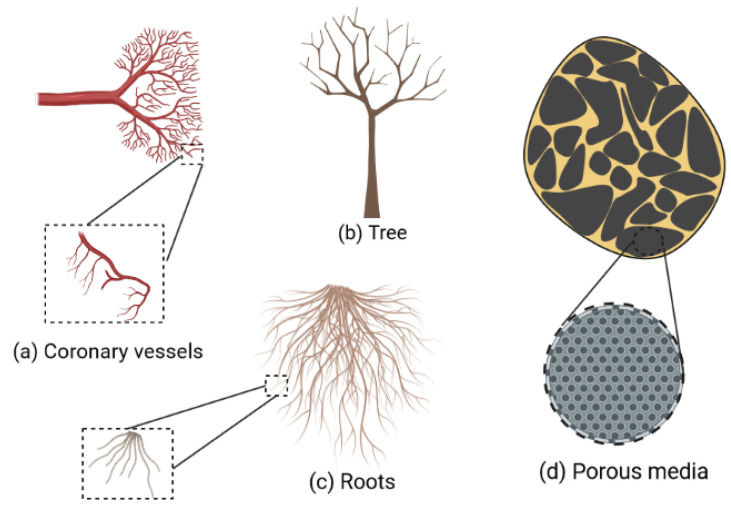
Examples of objects found in nature that exhibit a self-similar property.

A fractal is a type of complicated geometric shape that has a set of characteristics. Self-similarity, which can be described as the attribute of parts holding similarity to the whole at any level of magnification (see Figure 1), is one of the main characteristics of fractal objects [1]. Mandelbrot proposed fractional dimension as a property of fractals when he defined a fractal as a set with a Hausdorff dimension strictly greater than its topological dimension [2]. The fractal approach has triggered a surge of interest, and it has a wide range of practical applications in a variety of fields, including fluid mechanics [3,4,5,6], astronomy [7,8,9], acoustics [10,11,12,13], image analysis [14,15], geology and earth sciences [16,17,18,19], biology, and medicine [20,21,22,23,24,25].

**Figure 2:**
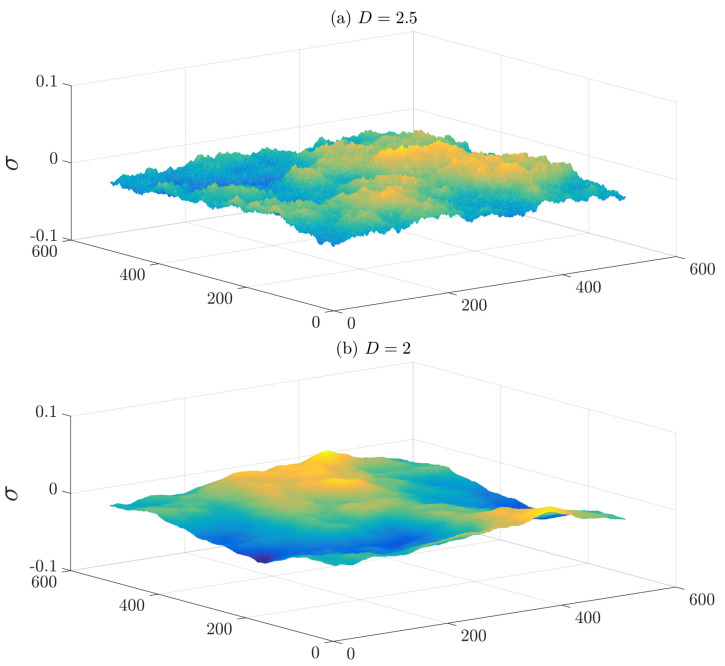
Two examples of fractal rough surfaces generated numerically. The root-mean-square surface height is the same for both surfaces, but the surface fractal dimensions are different.

The fractal approach can be used to study fluid flow through rough-walled pipes. A rough surface incorporates irregularities of varying sizes that have a special “scaling” connection with one another. They appear to fall within a regular hierarchy in which each level is a larger or smaller version of the level below or above it [26]. The scaling structure of a surface is described by a number D, referred to as the fractal dimension, which can range from two when the surface is smooth, and up to three. According to Brown [27], the surface fractal dimension and the ratio of the mean separation between the surfaces to the root-mean-square surface height are the major parameters determining the flow of a fluid through a medium with a rough structure. The former controls the asperity height while the latter scales the roughness of the surface (roughness thickness) (see Figure 2). For simplification, only the effects of the surface fractal dimension are considered in this study. This assumption provides accurate results in the following cases; when the fluid flow through a rough-walled pipe is controlled only by the surface fractal dimension, and when the ratio of the average pipe radius to the root-mean-square surface height is large enough.

The fractal dimension of a rough surface can be calculated by different methods, namely, the box-counting method, the PSD method, the roughness–length method and variogram method [28]. To model the effect of surface roughness on fluid flow using the fractal approach, several studies have been conducted [27,29,30,31,32,33,34]. In this paper, we consider the approach developed by Ghanbarian et al. [32], who established a power–law relationship between the length and radius of a rough pore. They investigated the flow of a Newtonian fluid through a rough pore-solid interface. The effects of the surface roughness on a power–law fluid flow using the tube length–radius relationship has not been considered. In this regard, this study presents a theoretical investigation of the effects of the surface roughness using fractal dimensions on the flow of a non-Newtonian fluid. We investigate the effect of the surface roughness on the velocity profile and the Darcy friction factor of an incompressible non-Newtonian fluid. Non-Newtonian fluids cover a wide range of applications [35,36,37,38]. Their flow patterns are frequently more complex than Newtonian fluids. Numerous research and observations have shown a non-linear relationship between viscous shear stress and velocity gradient in non-Newtonian fluids such as muddy clay, oils, blood, paints, and polymeric solutions. Several empirical or semi-empirical formulas, such as the well-known power–law model, the Bingham model, and the Casson model, have been proposed to accurately quantify non-Newtonian viscosity characteristics. In this study, the fluid is modeled using the well-known Ostwald–de Waele relationship (power–law) [39], where the shear stress τ is proportional to the shear rate $\dot{\gamma }$ to the power of the fluid constant *n*. Because of its simplicity, this mathematical relationship is useful, although it only approximates the behavior of a real non-Newtonian fluid. Other models exist that better describe the overall flow behavior of shear-dependent fluids, but they come at the sacrifice of simplicity. Therefore, the power–law is still useful to describe fluid behavior, enable mathematical predictions, and correlate experimental results [40,41,42]. For simplification, we assume the pipe to be axially symmetric. Although a more realistic case would be to consider an asymmetric pipe [35], this assumption is still useful for the case where the pipe has cylindrical symmetry, for example in Refs. [43,44,45]. This approach has a wide range of potential applications in any field that involves the flow of a non-Newtonian fluid through a tube with a “fractal” structure.

It is also crucial for the investigation of blood flow in the cardiovascular system [46,47,48]. Blood is considered to be a shear thinning non-Newtonian fluid, whose apparent viscosity decreases with increasing stress [49,50]. Furthermore, blood vessel walls are not smooth and regular in shape. It is well-known that the inner layer of an artery (tunica intima) is made up of endothelial cells, which are in direct contact with blood flow and can have a “fractal” structure [51,52]. Indeed, the rough structure of a pipe wall significantly influences the fluid flow and cannot be neglected.

The rest of this paper is organized in the following manner. In Section 2, we introduce the well-known Ostwald-de Waele relationship to model the rheological behavior of the fluid, the pipe it flows through being considered smooth. Section 3 deals with the fractal approach that describes the the pipe wall roughness and its effect on the behavior of the flow. In Section 4 an investigation and a discussion of the results obtained are presented. Finally, in Section 5, we present an overall conclusion about the results obtained.

## 2. Laminar Flow of a Non-Newtonian Fluid through a Pipe with a Smooth Surface

**Figure 3:**
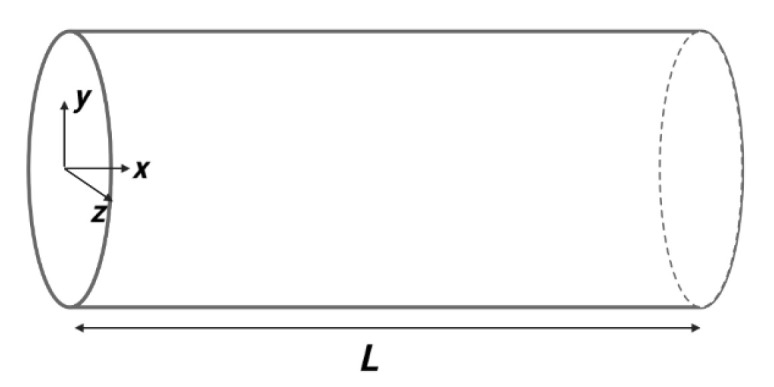
Schematic of a cylindrical pipe having a smooth surface.

Consider a steady laminar flow of a non-Newtonian fluid with constant properties through a pipe of radius *R* and length *L* (see Figure 3).

The rheological behavior of the fluid is modeled using the Ostwald-de Waele relationship, better known as the power–law model. The flow is assumed to be axially symmetric and fully developed. Modeling the hydrodynamics of any fluid requires the conservation of mass and the conservation of momentum equations [53]. These equations can be written in the following form:

(1) $$\frac{\partial }{\partial t}\;\int \int \int_{\;V}\rho dV=-\underset{S}{\;\int \int \;}\rho v.dS,$$

(2) $$\frac{\partial }{\partial t}\;\int \int \int_{\;V}\rho vdV=-\;\int \int_{\;S}\left(\rho v.dS\right)v-\;\int \int_{\;S}pdS+\;\int \int \int \;\rho f_{body}dV+F_{surf},$$

where v is the velocity field, *p* the pressure of the fluid, ρ the density of the fluid, $f_{body}$ is a vector representing body forces, $F_{surf}$ is a vector representing surface forces, and *t* is time. The following is the differential form of the mass and the momentum conservation equations:

(3) $$\frac{\partial \rho }{\partial t}+\nabla .\left(\rho v\right)=0,$$

(4) $$\rho \frac{Dv}{Dt}=\rho f+\nabla .\overset{=}{\sigma },$$

where $\overset{=}{\sigma }$ is the stress tensor characterizing the rheological behavior of the fluid.

The stress tensor can be expressed as follows:

(5) $$\overset{=}{\sigma }=-p\overset{=}{I}+\overset{=}{\tau },$$

where *p* and $\overset{=}{I}$ denote the pressure and unit tensor, respectively. $\overset{=}{\tau }$ refers to the shear tensor.

For a fully developed flow of a non-Newtonian incompressible fluid through a unidirectional cylindrical pipe, the conservation equations of mass and momentum (3) and (4) take the following form:

(6) $$\nabla .\left(\rho v\right)=0$$

(7) $$-\nabla p+\nabla .\overset{=}{\tau }=0.$$

Using the Ostwald-de Waele relationship [39], the shear tensor in Equation (7) takes the following expression:

(8) $$\overset{=}{\tau }=K_{0}{\left(\bar{\bar{\dot{\gamma }}}\right)}^{n},$$

where *n* and $K_{0}$ are empirical constants specific to the fluid type. $\bar{\bar{\dot{\gamma }}}$ denotes the strain rate tensor. For a Newtonian fluid we have n=1 and $K_{0}=\mu$, where μ denotes viscosity.

For a unidirectional flow (along the *x* direction in Figure 3), the projection of Equation (7) according to the *r* radial coordinate gives:

(9) $$\nabla_{r}\left(-\tau_{xr}e_{x}\right)=\frac{dp}{dx}e_{x}$$

where $\nabla_{r}$ denotes the divergence operator in the radial direction. Note that to maintain the flow in the positive *x* direction, the pressure gradient dp/dx must be negative.

Poiseuille’s flow differs from flows with inertia in that the pressure field is independent of the velocity field. Therefore, Equation (9) is written as follows:

(10) $$\frac{1}{r}\frac{d}{dr}\left(r\tau_{xr}\right)=\frac{\Delta p}{L},$$

where Δp>0 is the pressure difference along the pipe, and *L* is the pipe length.

The integration of Equation (10) between r=0 and r=R, taking into account the cylindrical symmetry of the pipe at r=0, makes it possible to express the wall shear stress as follows:

(11) $$\tau_{w}=\frac{\Delta p}{L}\frac{R}{2}.$$

By replacing the expression of the wall shear stress in Equation (10), we obtain:

(12) $$\frac{1}{r}\frac{d}{dr}\left(rK_{0}{\dot{\gamma }}_{xr}^{n}\right)=\frac{2\tau_{w}}{R}.$$

The shear rate ${\dot{\gamma }}_{xr}$ acts opposite to the direction of the flow (viscous friction). In fact, the shear rate is written as follows:

(13) $${\dot{\gamma }}_{xr}=-\frac{dv_{x}}{dr},$$

where $v_{x}=v_{x}\left(r\right)$ is the velocity component in the *x* direction, which is dependent on *r* only because of the symmetry of the pipe.

The integration of Equation (12), taking into account Equation (13), gives:

(14) $$\frac{dv_{x}}{dr}=-{\left(\frac{\tau_{w}}{K_{0}R}\right)}^{\frac{1}{n}}r^{\frac{1}{n}}.$$

The solution of Equation (14), considering the no-slip condition at the wall, makes it possible to obtain an analytical expression of the velocity field. Regarding the boundary condition at the wall, the reader should note that there are two schools of thought. The classic argument is that because most surfaces are rough, viscous dissipation when fluid travels past surface irregularities causes it to stop [54]. This has been challenged by evidence suggesting that when molecularly smooth surfaces are only partially wet, hydrodynamic models produce better results when “partial slip” boundary conditions are used instead [55,56]. Both ideas can be used to describe data, however, under certain limits (see Ref. [57] for more details). Moreover, Koplik et al. [58] studied the flow of a fluid on a plate using molecular dynamics. For gases, when the dimensions of the pipe are of the order of magnitude of the mean free path of a molecule, the no-slip boundary condition is no longer valid. However, for the case of a liquid, the results have demonstrated that the flow can still be considered as a continuum and the Navier–Stokes equations are still valid in this case. Therefore, it is the no-slip boundary condition. The velocity profile of a power–law fluid has the following form:

(15) $$v_{x}=v_{max}\left(n\right)\left[1-{\left(\frac{r}{R}\right)}^{\frac{1+n}{n}}\right],$$

where:

(16) $$v_{max}\left(n\right)={\left(\frac{\Delta pR}{2K_{0}L}\right)}^{\frac{1}{n}}\frac{nR}{1+n}.$$

Notice that for the case of a Newtonian fluid (n=1 and $K_{0}=\mu$), we obtain the usual Poiseuille equation:

(17) $$v_{x}=\frac{1}{4\mu }\frac{\Delta p}{L}R^{2}\left[1-{\left(\frac{r}{R}\right)}^{2}\right].$$

**Figure 4:**
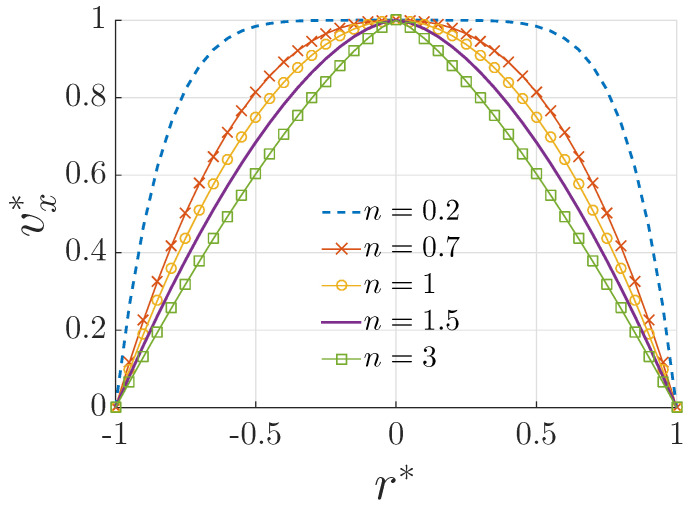
Plot of velocity profile defined by Equation (15) for different values of *n*, with $r^{*}=r/R$, $v_{x}^{*}=v_{x}/v_{max}\left(n\right)$.

Equation (15) is a well-known velocity distribution that describes the rheological behavior of non-Newtonian fluids. The fluid is shear thinning for 0<n<1, Newtonian for n=1 and shear thickening for n>1. These types of fluids are depicted in Figure 4, which plots the normalized velocity profile $v_{x}^{*}=v_{x}/v_{max}\left(n\right)$, where $v_{x}$ is defined by Equation (15) for different values of *n*. The normalized expression $v_{x}^{*}$ is used so we can see the influence of the power–law index (n) on the shape of the velocity profile. Shear thinning (0<n<1) is the non-Newtonian behavior of fluids whose viscosity decreases under shear strain. It is sometimes used interchangeably with the term “pseudo plastic behavior” [59,60]. It is the most common sort of non-Newtonian fluid behavior and is observed in numerous industrial and everyday applications [61]. Although the precise cause of shear thinning is not completely understood, it is broadly accepted as being the impact of little changes inside the fluid, such that microscale geometries inside the fluid rearrange to facilitate shearing. A shear thickening or dilatant material (n>1), is a material whose viscosity increases when shear stress is applied. The observed behavior of dilatant fluids is due to the system crystallizing under stress and acting more as a solid rather than as a solution [62].

In what comes next, we use the Ghanbarian et al. model [32] to investigate the effects of the tube wall roughness on the flow of a non-Newtonian fluid.

## 3. Laminar Flow of a Non-Newtonian Fluid through a Rough-Walled Pipe

In most natural cases, tubes such as pores in a porous medium or a blood vessel do not have a regular and smooth structure. To some degree they often have a rough surface and irregular cross-section. The geometric shape of a rough surface can be modeled using surface fractal dimension. The latter is used to quantify the infinitesimally self repeating irregularities present in the walls of a rough tube. Because of significant pressure loss and changes in velocity profile, flow in micron-scale tubes is more complicated than flow in millimeter-scale or larger tubes, especially when the tube wall is rough. The velocity profile is highly affected by the surface roughness, which increases the pressure drop. This has been looked into in ref [30,63,64], where it was discovered that roughness affects the velocity distribution of laminar Newtonian fluid flow in microchannels, resulting in a significant pressure drop throughout the channel length.

**Figure 5:**
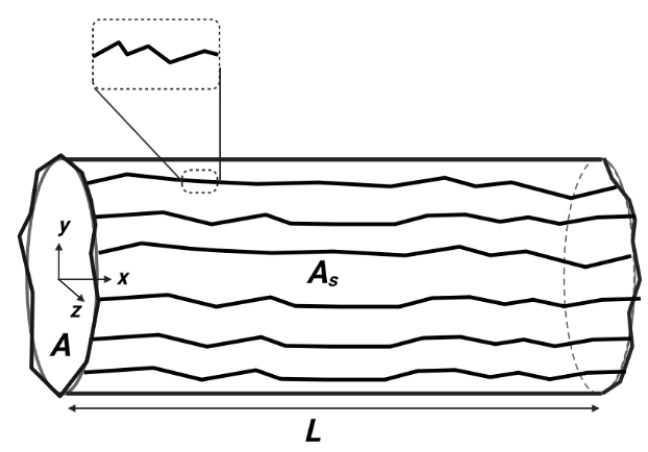
Schematic of a rough-structured tube.

**Figure 6:**
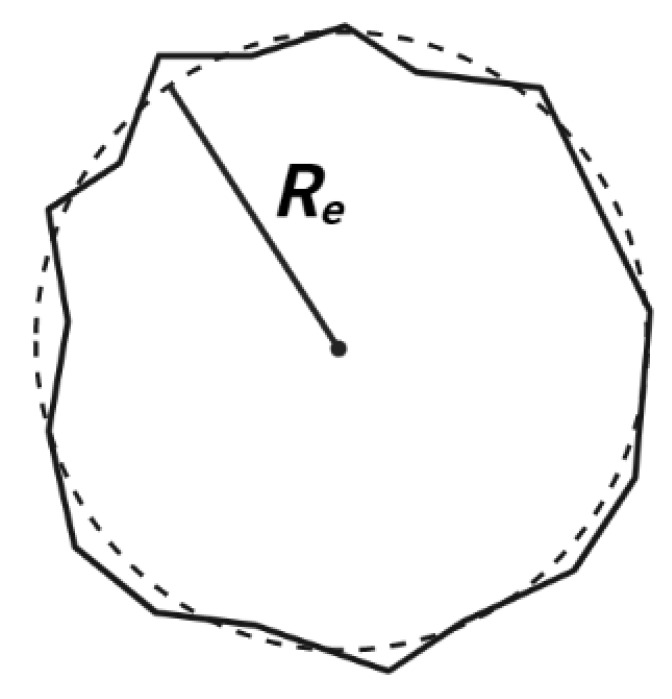
Scematic of an irregular cross section.

Let us consider an incompressible non-Newtonian fluid flow through a tube having a rough structure (see Figure 5). A tube having a rough structure is considered to be a fractal object, and can be modeled using the model developed by Ghanbarian et al. [32], who proposed a relationship between the tube equivalent radius $R_{e}$ (see Figure 6) and its length *L*. According to Mandelbrot [2], Mandelbrot et al. [26] and Lovejoy [65], the perimeter P of a fractal object can be related to its cross-sectional area A as follows:

(18) $$P^{2}\propto A^{D_{s2}},$$

where $D_{s2}$ is the surface fractal dimension $\left(1\leq D_{s2}<2\right)$, which measures the roughness of the cross sectional area of the tube. It is worth noting that Equation (18) has been experimentally tested in soils [66] and rocks [67]. For a smooth surface $D_{s2}$ equals 1, and when $D_{s2}$ approaches 2 the surface becomes rougher (or fractal). The cross sectional area *A* is simply approximated as $A\propto R_{e}^{2}$, where $R_{e}$ is an average radius (see Figure 6).

If the structure of a fractal oibject is invariant by an isotropic rescaling of lengths, it is (statistically) self-similar. This means that the same structure is recreated when lengths in different directions are rescaled with the same scaling factor [68]. As a result, at all scales, a (statistically) self-similar fractal seems the same. However, in nature different scaling exponents (e.g., fractal dimensions) and factors in different directions are used to scale fractal objects. This form of scale-invariance suggests that the fractal object is structurally anisotropic and self-affine [69], and that it cannot be characterized by just one fractal dimension.

The roughness of a tube can also be described by the surface fractal dimension $D_{s3}$ in three dimensions $\left(2\leq D_{s3}<3\right)$ [32]. The dimension $D_{s3}$ describes the roughness of the cross section along the tube structure (see Figure 5). The surface becomes exceedingly rough as $D_{s3}$ approaches 3, and $D_{s3}=2$ symbolizes a smooth surface. According to Mandelbrot [2] and Mandelbrot et al. [26], the relationship between a rough-structured tube surface area $A_{s}$ and its volume *V* (see Figure 5) for a fractal object is:

(19) $$A_{s}^{3}\propto V^{D_{s3}},$$

where

**Figure 7:**
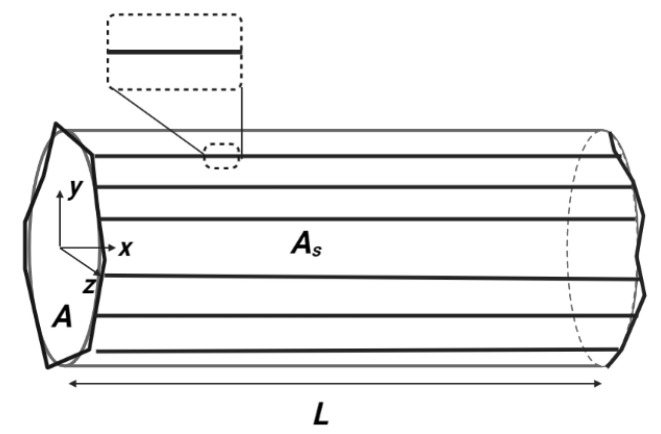
Schematic of a tube structure with $D_{r}=1$.

(20) V=A×L,

where *L* is the tube length. The surface area $A_{s}$ can be approximated as follows:

(21) $$A_{s}\propto P\times L^{D_{r}},$$

where the fractal length $L_{f}=L^{D_{r}}$ is proportional to the straight line *L* to the power of $D_{r}$. This latter is the roughness fractal dimension, which describes the roughness of a line along the tube length (*x* direction in Figure 5). Note that for $D_{r}=1$ the lines in the tube structure along the *x* direction become straight and Figure 5 becomes Figure 7.

Equations (20) and (21) are combined with Equations (18) and (19) to produce the following relationship:

(22) $$L\propto R_{e}^{\frac{2D_{s3}-3D_{s2}}{3D_{r}-D_{s3}}},$$

$Dr=D_{s3}-1$ if the roughness along the pipe structure is assumed to be isotropic [2,68]. In this case Equation (22) becomes:

(23) $$L\propto R_{e}^{\Gamma },$$

where $\Gamma =\frac{2D_{s3}-3D_{s2}}{2D_{s3}-3}$. Since $1\leq D_{s2}<2$ and $2\leq D_{s3}<3$, the permitted values of Γ range between −2 and 1. Equation (23), which requires two fractal dimensions $D_{s2}$ and $D_{s3}$ to characterize the tube roughness in different directions, is true for anisotropic fractal media [32]. In a (statistically) isotropic self-similar fractal medium [32], one can set

$$D_{s2}=D_{r}=D_{s3}-1.$$

As a result, in such a medium one gets:

(24) $$\Gamma =\frac{3-D_{s3}}{2D_{s3}-3}.$$

In a self-similar isotropic fractal object, Γ ranges between 1 to 0, demonstrating a direct link between the tube length and radius.

The proportionality constant in the relationship between *L* and $R_{e}$ (Equation (23)) has no effect on the shape of the velocity profile but does change the amplitude. Therefore, we set:

(25) $$L=c\times R_{e}^{\Gamma },$$

where *c* is a geometry constant.

### 3.1. Velocity Profile

We can now apply the previously-established relationship between *L* and the average radius $R_{e}$ to describe a non-Newtonian fluid flow though a channel having a rough surface. Relplacing Equation (25) in the wall shear stress expression (11) yields:

(26) $$\tau_{w}=\frac{\Delta p}{cR_{e}^{\Gamma }}\frac{R}{2}=\frac{\Delta p}{2c}R_{e}^{1-\Gamma }.$$

The velocity profile of a non-Newtonian fluid flow through a rough-walled tube is:

(27) $$v_{x}\left(r\right)=v_{max}\left(n,\Gamma \right)\left[1-{\left(\frac{r}{R_{e}}\right)}^{\frac{1+n}{n}}\right],$$

where:

(28) $$v_{max}\left(n,\Gamma \right)=\frac{n}{1+n}{\left(\frac{\Delta p}{2cK_{0}}\right)}^{\frac{1}{n}}R_{e}^{\frac{n+1-\Gamma }{n}}.$$

Note that we have replaced $R\to R_{e}$, because the cross section of a rough tube is generally irregular (see Figure 6). Hence, it is more convenient to use an equivalent radius. Equation (27) can be used to describe a non-Newtonian fluid flow through a rough-structured pipe. For the case of a Newtonian fluid (n=1, $K_{0}=\mu$) and a smooth surface (Γ=1), we obtain the usual Poiseuille equation:

(29) $$v_{x}\left(r\right)=\frac{1}{4\mu }\frac{\Delta p}{L}R^{2}\left[1-{\left(\frac{r}{R}\right)}^{2}\right].$$

### 3.2. Darcy Friction Factor

According to the Darcy–Weisbach equation [70], the friction factor can be written in the following expression:

(30) $$f=\frac{8\tau_{w}}{\rho {\bar{v}}^{2}}$$

where $\tau_{w}$ is the wall shear stress defined by Equation (11), ρ the fluid density, and $\bar{v}$ represents the fluid mean velocity. It can be obtained using the velocity profile defined by Equation (15), where:

(31) $$\bar{v}=\frac{1}{A}\int_{0}^{R}v_{x}\left(r\right)2\pi rdr=\frac{n}{3n+1}{\left(\frac{\Delta p}{2K_{0}L}\right)}^{\frac{1}{n}}R^{\frac{n+1}{n}},$$

where $A=\pi R^{2}$ is the cross-sectional area.

Substituting the wall shear stress expression (11) and Equation (31) into Equation (30) yields:

(32) $$f=\frac{8\tau_{w}}{\rho {\bar{v}}^{2}}=\frac{8{\left(3n+1\right)}^{2}}{\rho n^{2}}{\left(K_{0}\right)}^{\frac{2}{n}}{\left(\frac{\Delta p}{2L}\right)}^{\frac{n-2}{n}}R^{-\frac{n+2}{n}}.$$

The above equation represents the friction factor for a non-Newtonian fluid flowing through a tube having a smooth surface. The case of a fractal (rough) surface can be easily obtained by substituting the length–radius relationship (25) into Equation (32), which yields:

(33) $$f=GR^{\frac{m-4}{n}},$$

where:

$$G=\frac{8{\left(3n+1\right)}^{2}}{\rho n^{2}}{\left(K_{0}\right)}^{\frac{2}{n}}{\left(\frac{\Delta p}{2c}\right)}^{\frac{n-2}{n}}$$

m=(1+Γ)(2−n).

Equation (33) represents the Darcy friction factor that describes friction losses in a rough-structured pipe flow. For the case of a smooth surface (Γ=1) and a Newtonian fluid (n=1, $K_{0}=\mu$), we obtain the well known expression for the friction factor:

(34) $$\begin{array}{c} f=\frac{64}{ℜe}, \end{array}$$

where ℜe is the Reynolds number:

$$ℜe=\frac{\rho D\bar{v}}{\mu }.$$

## 4. Results and Discussion

**Figure 8:**
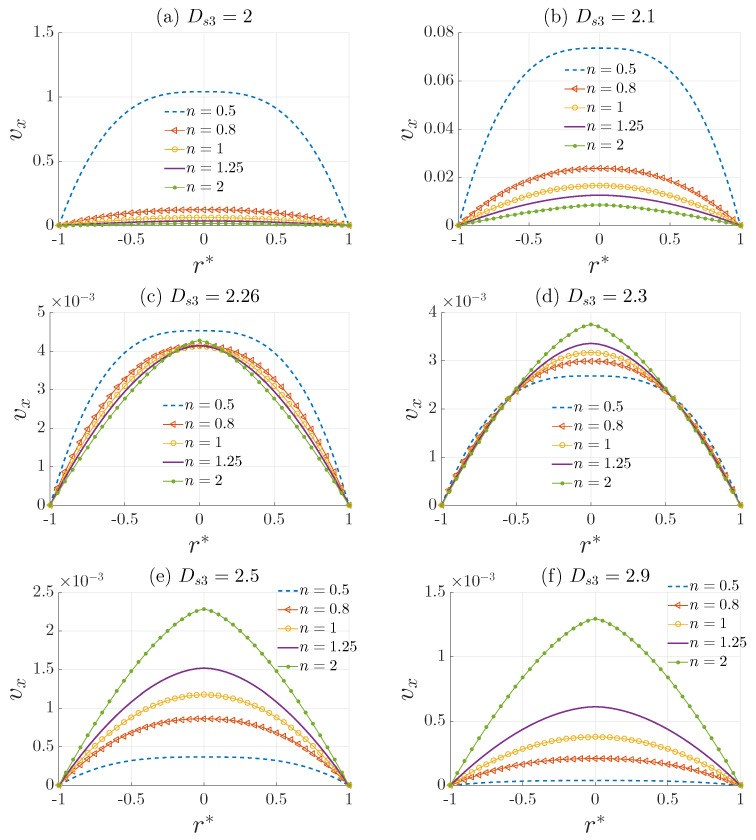
Influence of the surface fractal dimension $D_{s3}$ on the velocity profile $v_{x}$ (m/s) defined by Equation (27) for different values of *n*, with $r^{*}=r/R_{e}$, $R_{e}=5$ mm, Δp=5 Pa, $K_{0}=10^{-3}$ Pa·s^n^, and c=100 m^1−Γ^.

**Figure 9:**
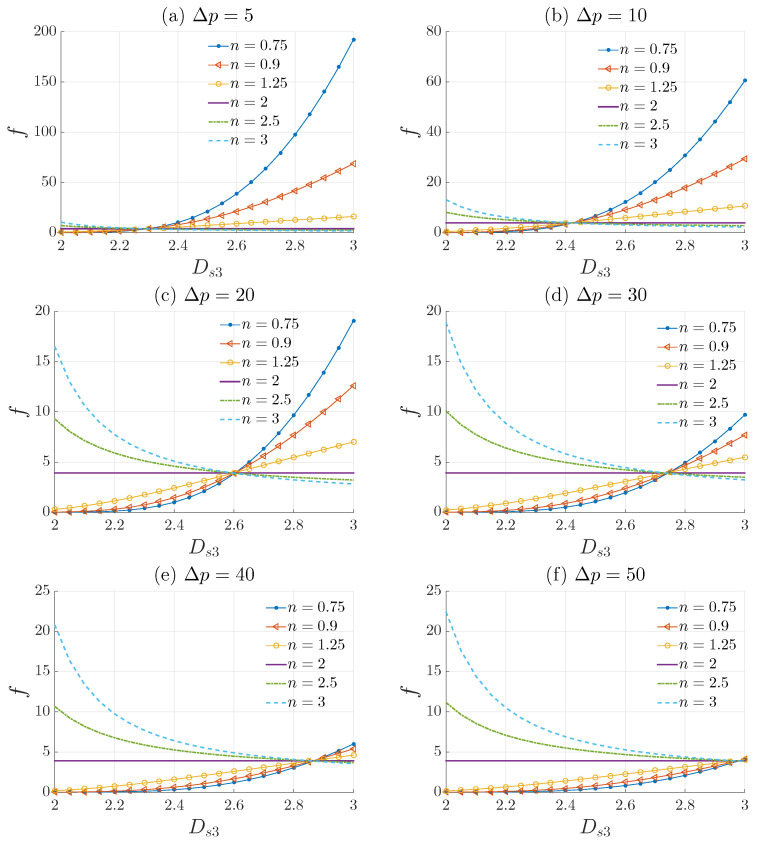
Plot of the friction factor defined by Equation (33) with respect to $D_{s3}$ for different values of *n* with $R_{e}=5$ mm, Δp=[5,10,20,30,40,50] Pa, ρ=1000 kg/m^3^, $K_{0}=10^{-3}$ Pa.s^n^ and c=100 m^1−Γ^.

In the fully developed laminar flow of a viscous power–law fluid through a rough-structured tube, the power–law index (*n*) and the surface fractal dimension ($D_{s3}$) have a significant effect on the flow behavior determined by the velocity distribution and the friction factor. The rough structure of the tube is described using the length–radius relationship $L\propto R_{e}^{\Gamma }$. For an isotropic fractal medium, $\Gamma =\left(3-D_{s3}\right)/\left(2D_{s3}-3\right)$, where only one dimension is needed to measure the roughness of the tube surface. When the medium is anisotropic, modern approaches such as 3-D image analysis should be used to calculate the roughness exponents (e.g., $D_{s2}$ and $D_{s3}$). The effects of the surface fractal dimension $D_{s3}$ are depicted in Figure 8 and Figure 9.

Figure 8 illustrates the influence of the roughness of the pipe surface on the velocity profile defined by Equation (27). It is noteworthy that the surface fractal dimension does not affect the shape of the velocity profile, but only the amplitude. Therefore, in this case a dimensional velocity is used to visualize the effects of surface roughness. We can see that as $D_{s3}$ increases the amplitude of the velocity profile decreases, which is expected since $D_{s3}$ is a measure of the surface roughness. Accordingly, an increase in the dimension $D_{s3}$ results in an increase in friction losses. However, the rate of decrease in the velocity profile amplitude differs for different values of *n*. A faster decrease can be observed for shear thinning fluids, where n<1 compared with Newtonian n=1 and shear thickening fluids n>1. Consequently, it is safe to assume that shear thinning (pseudo-plastic) fluids are more sensitive to the surface roughness. Shear-thinning is a phenomenon characteristic of fluids such as blood, motor oil, ketchup, and even whipped cream in which the fluid viscosity decreases with increasing shear stress. Therefore, with increasing roughness, the level of viscosity is lower, which makes shear-thinning fluids more sensible to the surface roughness. We can also see that for a particular value of $D_{s3}\approx 2.26$, the amplitude of the velocity profile for all values of *n* is approximately the same.

This phenomenon is also observed in Figure 9, which plots the variation of the friction factor defined by Equation (33) with respect to $D_{s3}$ for different values of *n* and Δp. We can see that for a particular value of $D_{s3}=D_{cr}$, the friction factor for different values of *n* is approximately the same. The critical value $D_{cr}$ represents a point of intersection where for all values of *n* the friction factor and the amplitude of the velocity profile are the same. Moreover, the value of $D_{cr}$ is not fixed. It is heavily dependent on the flow characteristics, that is, it changes for different values of Δp. In Figure 9 we have for (a) $D_{cr}\approx 2.25$, (b) $D_{cr}\approx 2.42$, (c) $D_{cr}\approx 2.6$, (d) $D_{cr}\approx 2.75$, and (e) $D_{cr}\approx 2.88$, and for (f) the intersection point does not exist. This means that there are two regimes that can be distinguished. The first regime is where Δp<50 Pa, the critical point $D_{cr}$ exists, and the second regime is where Δp>50 Pa, the critical point $D_{cr}$ does not exist. It should be noted that the pressure drop Δp is not the only influencing factor. The radius $R_{e}$, the empirical constant $K_{0}$, and the geometry constant *c* all have an influence on the values of $D_{cr}$. Figure 9 also demonstrates that for fluids with n<2 the friction factor increases, contrary to fluids with n>2. Fluids with n=2 are not affected by the surface roughness, as we can see from Figure 9, regardless of the values of $D_{s3}$, the friction factor for a fluid with n=2 remains constant:

(35) $$f=\frac{72K_{0}}{\rho R^{2}}.$$

Shear-thickening fluids are less sensible to the surface roughness because with increasing shear stress, their viscosity increases. Thus, n=2 represents a critical value where for n>2 the surface roughness starts to have opposite effects (Friction factor decreases). The strange behavior of shear thickening fluids with n≥2 needs to be experimentally investigated in future studies in order to know the exact interpretation of these results. It is also important to note that for fluids with n>2 and $D_{s3}>D_{cr}$, the friction factor tends towards a constant value. By contrast, for n<2 and $D_{s3}>D_{cr}$, the friction factor increases significantly, especially for shear thinning fluids (n<1). This explains the impact the surface fractal dimension has on the amplitude of the velocity profile of shear thinning fluids.

## 5. Concluding Remarks

In this study, a theoretical investigation of the effects of the pipe surface roughness on the velocity profile and the Darcy friction factor of a non-Newtonian fluid was presented.

The rheological behavior of the fluid was modeled using the Ostwald–de Waele relationship, and the roughness of the pipe was quantified using surface fractal dimensions. Next, we obtained new analytical expressions for the velocity profile and the friction factor of a power–law fluid. Because of their nature, shear thinning fluids were the most affected by the tube surface roughness, compared with Newtonian and shear thickening fluids. Regardless of the values of the power–law index (*n*), for a particular value ($D_{cr}$) of the surface fractal dimension ($D_{s3}$), the values of the friction factor are approximately the same, and this is true for the amplitude of the velocity profile as well. $D_{cr}$ depends on the pressure drop Δp, the pipe’s equivalent radius $R_{e}$, the empirical constant $K_{0}$, and the geometry constant *c*.

Finally, we anticipate that our findings can be applied for various industrial applications that involve non-Newtonian fluids flows through axisymmetric rough-walled tubes, fluid dynamics in hydrology or blood flow in the cardiovascular system.The results obtained in this study are mainly theoretical, and further experimental investigations to study real samples of rough surfaces are considered in future studies.

## Data Availability

Not applicable.

## References

[1] Addison P.S. (1997). Fractals and Chaos: An Illustrated Course.

[2] Mandelbrot B.B. (1982). The Fractal Geometry of Nature.

[3] Tarasov V.E. (2016). Poiseuille equation for steady flow of fractal fluid. Int. J. Mod. Phys..

[4] Balankin A.S., Mena B., Susarrey O., Samayoa S. (2017). Steady laminar flow of fractal fluids. Phys. Lett..

[5] Akgül A., Siddique I. (2021). Novel applications of the magnetohydrodynamics couple stress fluid flows between two plates with fractal-fractional derivatives. Numer. Methods Partial. Differ. Equ..

[6] Xie J., Gao M., Zhang R., Liu J., Liu Y., Yang B., Wang M., Wang F. (2021). Fluid flow characteristics of cross-fractures with two branch fractures of different roughness controlled by fractal dimension: An experimental study. J. Pet. Sci. Eng..

[7] Ortiz C.H., Cortes E.S., Bonilla J.L., Castañeda H. (2015). Fractal di- mension and turbulence in giant hii regions. J. Phys. Conf. Ser..

[8] Gaite J. (2019). The fractal geometry of the cosmic web and its formation. Adv. Astron..

[9] Lancaster L., Ostriker E.C., Kim J.G., Kim C.G. (2021). Efficiently Cooled Stellar Wind Bubbles in Turbulent Clouds. I. Fractal Theory and Application to Star-forming Clouds. Astrophys. J..

[10] Berbiche A., Fellah M., Fellah Z.E.A., Ogam E., Mitri F.G., Depollier C. (2017). Transient acoustic wave in self-similar porous material having rigid frame: Low frequency domain. Wave Motion.

[11] Fellah M., Fellah Z.E.A., Berbiche A., Ogam E., Mitri F.G., Depollier C. (2017). Transient ultrasonic wave propagation in porous material of non-integer space dimension. Wave Motion.

[12] Fellah Z.E.A., Fellah M., Ogam E., Berbiche A., Depollier C. (2021). Reflection and transmission of transient ultrasonic wave in fractal porous material: Application of fractional calculus. Wave Motion.

[13] Fellah Z.E.A., Fellah M., Ongwen N.O., Ogam E., Depollier C. (2021). Acoustics of fractal porous material and fractional calculus. Mathematics.

[14] Soille P., Rivest J.F. (1996). On the validity of fractal dimension measurements in image analysis. J. Vis. Commun. Image Represent..

[15] Guariglia E. (2019). Primality, fractality, and image analysis. Entropy.

[16] Av¸sar E. (2020). Contribution of fractal dimension theory into the uniaxial compressive strength prediction of a volcanic welded bimrock. Bull. Eng. Geol. Environ..

[17] Heping X. (2020). Fractals in Rock Mechanics.

[18] Ildoromi A. (2021). Separation of geological formations by comparing the density dimension of drainage network and fractal dimension of drainage network (Case study: Northern slopes of Hamedan). New Find. Appl. Geol..

[19] Tiwari R.K., Paudyal H. (2021). Box Counting Fractal Dimension and Frequency Size Distributon of Earthquakes in the Central Himalaya Region. J. Inst. Sci. Technol..

[20] Mambetsariev I., Mirzapoiazova T., Lennon F., Jolly M.E., Li H., Nasser M.W., Vora L., Kulkarni P., Batra S.K., Salgia R. (2019). Small cell lung cancer therapeutic responses through fractal measurements: From radiology to mitochondrial biology. J. Clin. Med..

[21] Pham D.T., Musielak Z.E. (2021). Spectra of Reduced Fractals and their Applications in Biology. arXiv.

[22] Szasz A. (2021). Time-Fractal in Living Objects. Open J. Biophys..

[23] Watanabe H., Hayano K., Ohira G., Imanishi S., Hanaoka T., Hirata A., Kano M., Matsubara H. (2021). Quantification of structural heterogeneity using fractal analysis of contrast-enhanced CT image to predict survival in gastric cancer patients. Dig. Dis. Sci..

[24] Soltani P., Sami S., Yaghini J., Golkar E., Riccitiello F., Spagnuolo G. (2021). Application of Fractal Analysis in Detecting Trabecular Bone Changes in Periapical Radiograph of Patients with Periodontitis. Int. J. Dent..

[25] Elkington L., Adhikari P., Pradhan P. (2022). Fractal Dimension Analysis to Detect the Progress of Cancer Using Transmission Optical Microscopy. Biophysica.

[26] Mandelbrot B.B., Passoja D., Paullay A.J. (1984). Fractal character of fracture surfaces of metals. Nature.

[27] Brown S.R. (1987). Fluid flow through rock joints: The effect of surface roughness. J. Geophys. Res. Solid Earth.

[28] Zhang X., Xu Y., Robert L.J. (2017). An analysis of generated fractal and measured rough surfaces in regards to their multi-scale structure and fractal dimension. Tribol. Int..

[29] Brown S.R., Stockman H.W., Reeves S.J. (1995). Applicability of the reynolds equation for modeling fluid flow between rough surfaces. Geophys. Res..

[30] Chen Y., Zhang C., Shi M., Peterson G.P. (2009). Role of surface roughness characterized by fractal geometry on laminar flow in microchannels. Phys. Rev. E.

[31] Wojciech M., Ricardo B., Mateusz K., Tadeusz L. (2021). Fractal dimension for bending–torsion fatigue fracture characterisation. Measurement.

[32] Ghanbarian B., Hunt A.G., Daigle H. (2016). Fluid flow in porous media with rough pore-solid interface. Water Resour. Res..

[33] Gancarczyk A., Sindera K., Iwanisyzn M., Piątek M., Macek W., Jodlowski P.J., Wroński S., Sitarz M., Łojewska J., Kołodziej A. (2019). Metal Foams as Novel Catalyst Support in Environmental Processes. Catalysts.

[34] Tang W., Wang Y. (2012). Fractal characterization of impact fracture surface of steel. Appl. Surf. Sci..

[35] Hayat T., Yasmin H., Alhuthali M.S., Kutbi M.A. (2013). Peristaltic Flow of a Non-Newtonian Fluid in an Asymmetric Channel with Convective Boundary Conditions. J. Mech..

[36] Hayat T., Yasmin H., Alsaedi A. (2015). Convective heat transfer analysis for peristaltic flow of power-law fluid in a channel. J. Braz. Soc. Mech. Sci. Eng..

[37] Yasmin H., Iqbal N., Hussain A. (2020). Convective heat/mass transfer analysis on Johnson-Segalman fluid in a symmetric curved channel with peristalsis: Engineering applications. Symmetry.

[38] Vikash P., Sverre H. (2016). Linking the fractional derivative and the Lomnitz creep law to non-Newtonian time-varying viscosity. Phys. Rev. E.

[39] Shapovalov V.M. (2017). On the applicability of the Ostwald–de Waele model in solving applied problems. J. Eng. Phys. Thermophys..

[40] Hussain S., Öztop H.F. (2021). Impact of inclined magnetic field and power law fluid on double diffusive mixed convection in lid-driven curvilinear cavity. Int. Commun. Heat Mass Transf..

[41] Saeed Khan M.W., Ali N. (2021). Thermal entry flow of power-law fluid through ducts with homogeneous slippery wall(s) in the presence of viscous dissipation. Int. Commun. Heat Mass Transf..

[42] Abu-Nab A.K., Selima E.S., Morad A.M. (2021). Theoretical investigation of a single vapor bubble during Al2O3/H2O nanofluids in power-law fluid affected by a variable surface tension. Phys. Scr..

[43] Oyelami F.H., Ige E.O., Taiyese N.O., Saka-Balogun O.Y. (2021). Magneto-radiative analysis of thermal effect in symmetrical stenotic arterial blood flow. J. Math. Comput. Sci..

[44] Haghighatkha A., Kahriz M.A. (2021). Numerical simulation of intravenous blood flow. J. Multidiscip. Eng. Sci. Technol..

[45] Pakhomov M.A., Zhapbasbayev U.K. (2021). RANS modeling of turbulent flow and heat transfer of non-Newtonian viscoplastic fluid in a pipe. Case Stud. Therm. Eng..

[46] Gabry’s E.Z., Rybaczuk M., K˛edzia A. (2006). Blood flow simulation through fractal models of circulatory system. Chaos Solitons Fractals.

[47] G Jayalalitha G., Deviha V.S., Uthayakumar R. (2008). Fractal model for blood flow in cardiovascular system. Comput. Biol. Med..

[48] Deviha V.S., Rengarajan P., Hussain R.J. (2013). Modeling blood flow in the blood vessels of the cardiovascular system using fractals. Appl. Math. Sci..

[49] da Silva J.L., Rao M.A. (2007). Rheology of Fluid and Semisolid Foods.

[50] Schramm L.L. (2005). Emulsions, Foams, and Suspensions Fundamentals and Applications.

[51] Wlczek P., Odgaard A., Sernetz M. (1992). Fractal Geometry and Computer Graphics.

[52] Chen C., Zhang X., Ren L., Geng Y., Bai G. (2021). Analysis of blood flow characteristics in fractal vascular network based on the time fractional order. Phys. Fluids.

[53] Kundu P., Cohen I.M., Dowling D.R. (2012). Fluid Mechanics.

[54] Richardson S. (1973). On the no-slip boundary condition. J. Fluid Mech..

[55] Dietrich E., Peter P., Mario L. (1990). Boundary condition for fluid flow: Curved or rough surfaces. Phys. Rev. Lett..

[56] Thompson P.A., Robbins M.O. (1990). Shear flow near solids: Epitaxial order and flow boundary conditions. Phys. Rev. A.

[57] Zhu Y., Granick S. (2002). Limits of the hydrodynamic no-slip boundary condition. Phys. Rev. Lett..

[58] Koplik J., Banavar J.R., Willemsen J.F. (1989). Molecular dynamics of fluid flow at solid surfaces. Phys. Fluids A Fluid Dyn..

[59] Stieger M. (2002). The rheology handbook-for users of rotational and oscillatory rheometers. Appl. Rheol..

[60] Singh R.P., Heldman D.R., Sun D.W. (2001). Introduction to Food Engineering.

[61] Barnes H.A., Fletcher Hutton J.F., Walters K. (1989). An Introduction to Rheology.

[62] Painter P.C., Coleman M.M. (2019). Fundamentals of Polymer Science: An Introductory Text.

[63] Hu Y., Werner C., Li D. (2003). Influence of three-dimensional rough- ness on pressure-driven flow through microchannels. J. Fluids Eng..

[64] Wang H., Wang Y. (2007). Influence of three-dimensional wall roughness on the laminar flow in microtube. Int. J. Heat Fluid Flow.

[65] Lovejoy S. (1982). Area-perimeter relation for rain and cloud areas. Science.

[66] Pachepsky Y., Yakovchenko V., Rabenhorst M.C., Pooley C., Sikora L.J. (1996). Frac- tal parameters of pore surfaces as derived from micromorphological data: Effect of long-term management practices. Geoderma.

[67] Schlueter E.M., Zimmerman R.W., Witherspoon P.A., Cook N.G.W. (1997). The fractal dimension of pores in sedimentary rocks and its influence on permeability. Eng. Geol..

[68] Sahimi M. (2011). Flow and Transport in Porous Media and Fractured Rock: From Classical Methods to Modern Approaches.

[69] Carr J.R. (1997). Statistical self-affinity, fractal dimension, and geologic interpretation. Eng. Geol..

[70] Brown G.O. (2003). Environmental and Water Resources History.

